# Assessment of left ventricular volumes and mass using single-breath-hold 3D k-t BLAST cine b-SSFP in comparison with multiple-breath-hold 2D cine b-SSFP

**DOI:** 10.1186/1532-429X-11-S1-O99

**Published:** 2009-01-28

**Authors:** Alessandro Palumbo, Giancarlo Messalli, Erica Maffei, Chiara Martini, Cosetta Saccò, Filippo Cademartiri

**Affiliations:** Ospedale Maggiore di Parma, Parma, Italy

**Keywords:** Public Health, Ejection Fraction, Strong Correlation, Medical System, Related Parameter

## Purpose

To compare performance of new single-breath-hold 3D k-t BLAST cine b-SSFP sequence for left ventricular volume and mass evaluation using multiple-breath-hold 2D cine b-SSFP as a reference standard. We also compared time-efficiency of the two sequences calculating scan time and reporting time.

## Methods

On a commercially available 1,5 T MR scan (Achieva, Philips Medical System), single-breath-hold 3D k-t BLAST cine b-SSFP sequence (3D-cine) and multiple-breath-hold 2D cine b-SSFP sequence (2D-cine) were performed in 46 patients referred to investigate different diseases. The global functional parameters, LV mass, scan time and report time were evaluated in each patient for both sequences. Differences between functional parameters and LV mass were made with a paired Student's T test; correlation between parameters was assessed with Pearson's correlation coefficient. A Bland-Altman analysis was used to investigate the limits of agreement between the measurements. Differences between time-efficiency related parameters were made with a paired Student's T test.

## Results

Functional parameters and mass were significantly different in the two sequences (p < 0.05) but a strong correlation was found for LV ejection fraction (r = 0.96) and good correlation for other functional parameters (r between 0.83 and 0.93). Scan time was significantly lower for 3D sequence, report time was significantly higher for 3D sequence.

## Conclusion

3D k-t BLAST sequence can be used to assess EF in patients who have poor compliance in performing multiple apnoeas and in patients who are not able to remain in the scanner for a long time. Conversely report time is significantly higher for 3D sequence.

